# Supplementary material for: Reduction in the Use of Some Herbicides Favors Nitrogen Fixation Efficiency in Phaseolus vulgaris and Medicago sativa
Source: Plants (Basel). 2023 Apr 10;12(8):1608. doi: 10.3390/plants12081608 (PMC10144682; doi:10.3390/plants12081608)
Supplement: Supplementary file 1 [file plants-12-01608-s001.zip › Table S1.pdf]

Table S1. Primer sequences of *Phaseolus vulgaris* genes used for qRT-PCR.

Gene	Oligonucleotide sequence	Gene ID
UBC9	Fw 5´- GCTCTCCATTTGCTCCCTGTT-3´<br>Rv 5´- TGAGCAATTTCAGGCACCAA-3´	*Phvul.006G110100*
NF-YA1 (HAP2)	Fw 5´- TACTTTGGCAATCCATCCTTG-3´<br>Rv 5´- AGACAGTTCGGTGCAGAAAGA3´	*Phvul.001G196800*
ENOD40	Fw 5´- GGGTCCTTACCCCTCACACT-3´<br>Rv 5´- TGTAGCCAAAGCCTCTCATCC-3´	*Phvul.002G064200*

Provided by Rodríguez et al. 2020 [60].

Reference

60 Rodríguez, S.; Correa-Galeote, D.; Sánchez-Pérez, M.; Ramírez, M.; Isidra-Arellano, M.C.; Reyero-Saavedra, M.R.; Zamorano-Sánchez, D.; Hernández, G.; Valdés-López, O.; Girard, L. A Novel OmpR-Type Response Regulator Controls Multiple Stages of the *Rhizobium Etli—Phaseolus Vulgaris* N2-Fixing Symbiosis. *Front. Microbiol.* **2020**, *11*, 3250.
