# Supplementary figures and images for: Reduction in the Use of Some Herbicides Favors Nitrogen Fixation Efficiency in Phaseolus vulgaris and Medicago sativa
Source: Plants (Basel). 2023 Apr 10;12(8):1608. doi: 10.3390/plants12081608 (PMC10144682; doi:10.3390/plants12081608)

Figure S1. Soil textural triangle.

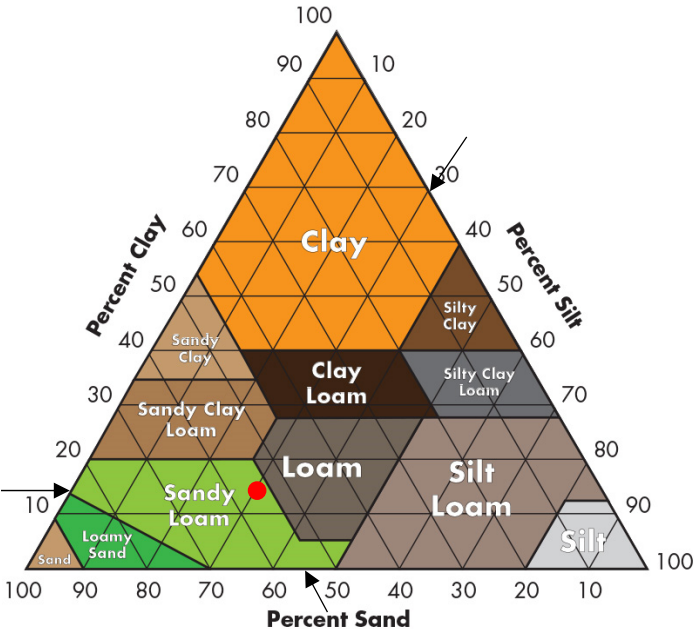

Supplement: Supplementary file 1 [file plants-12-01608-s001.zip › Supplementary Figure.pdf]
